# Supplementary material for: Perceptions of Medical Students Regarding Career Counseling in Korea: A Qualitative Study
Source: Int J Environ Res Public Health. 2020 May 16;17(10):3486. doi: 10.3390/ijerph17103486 (PMC7277443; doi:10.3390/ijerph17103486)
Supplement: Supplementary file 1 [file ijerph-17-03486-s001.pdf]

Supplementary Information

Perceptions of Medical Students Regarding Career Counseling in Korea: A Qualitative Study

Minsu Ock [1,2,†,*], Young-Joo Han [3,†,*], Eun Young Choi [1,4], Jeehee Pyo [1] and Won Lee [5]

[1] Department of Preventive Medicine, Ulsan University Hospital, University of Ulsan College of Medicine, Ulsan 44033, Republic of Korea; eesther0517@naver.com (J.P.)

[2] Department of Preventive Medicine, University of Ulsan College of Medicine, Seoul 05505, Republic of Korea

[3] Korea Counseling Graduate University, Seoul 06722, Republic of Korea

[4] Department of Nursing, Graduate School of Chung-Ang University, Seoul 06974, Republic of Korea; 11351@naver.com (E.Y.C);

[5] Department of Nursing, Chung-Ang University, Seoul 06974, Republic of Korea; oness38@daum.net (W.L.).

† These authors contributed equally to this work.

* correspondence: ohohoms@naver.com（M.O.）; hanyj@kcgu.ac.kr (Y.J.H.)

FGD guidelines for the career counseling of medical students

Ice-breaking(5-minute)

• Introduction and purpose explanation of the FGD

• Introduction of the FGD process

- Recording notification and approval confirmation
- Inform the FGD proceeding steps and precautions
- Inform about personal information protection

• Introduction of participants

Introductory/Overall Questions (20-minute)

1. The motivation of medical school admission

1) "What was your motivation for medical school enrollment?"

2) "Did you experience any challenges after the enrollment?"

Necessity Recognition of Career Counseling Program (30-minute)

2. Necessity recognition of career counseling program

1) "Assuming various challenges you could experience in medical school, what is the most difficult one you have experienced?"

▶ Investigative Question

- Identify the order of priority in previously mentioned challenges
- Identify the necessity status of career counseling

2) "How much have you contemplated on your career path?"

▶ Investigative Question

- Identify whether participants have conducted career exploration
- Identify whether participants have changed their opinions on their career
- "If you have not, what would be the reasons?" Identify the obstacles for career exploration (e.g., busy academic life, lack of information and more)
- "During your career exploration, who has influenced your research the most?"

3) "During your career exploration, what element has influenced your research the most?"

▶ Investigative Question

- Identify elements such as salary, working hour, quality of life, job satisfaction, and more

Opinions on the Development of Career Counseling Program (30-minute)

3. Opinions on the development of the career counseling program

1) "If a career counseling program is developed, would you like to participate?"

2) "If you were to design the content of a career counseling program, what would be the essential component for the program?"

3) "What form a career counseling program should have?"

4) "What is your thought on covering self-efficacy in a career counseling program?"

5) "Do you expect that people with a career counseling experience to choose their major well or to have a high job satisfactory?"

Other Discussion (5-minute)

• Additional and follow-up questions on the FGD

• Other questions

• Closing the discussion

- Gratitude demonstration on the FGD participation
